# Serum zinc concentration in patients with acute myocardial infarction in percutaneous coronary intervention era

**DOI:** 10.1371/journal.pone.0203074

**Published:** 2018-08-30

**Authors:** Toshitaka Okabe, Tadayuki Yakushiji, Suguru Shimazu, Jumpei Saito, Taro Kimura, Yuji Oyama, Wataru Igawa, Morio Ono, Takehiko Kido, Seitaro Ebara, Kennosuke Yamashita, Myong Hwa Yamamoto, Kisaki Amemiya, Naoei Isomura, Masahiko Ochiai

**Affiliations:** Showa University Northern Yokohama Hospital, Division of Cardiology and Cardiac Catheterization Laboratories, Yokohama, Japan; Osaka University Graduate School of Medicine, JAPAN

## Abstract

**Introduction:**

There were few studies that investigated the association between serum zinc concentration and acute myocardial infarction (AMI) in percutaneous coronary intervention era.

**Objective:**

We assessed the relationships between serum zinc concentration, complications, and prognosis in AMI patients after primary percutaneous coronary intervention.

**Methods:**

We conducted a single-center, prospective, observational study including 50 patients with AMI. We divided patients into two groups (High-zinc group and Low-zinc group) by median serum zinc concentration and compared two groups about clinical outcomes up to 1 year follow up.

**Results:**

The mean age of patients was 66.2 ± 11.8 years old. Patients in the Low-zinc group had ST-segment elevation more frequently than those in the High-zinc group (96.0% vs. 72.0%, P = 0.02). All-cause mortality at 1 year was similar in both groups (P (log-rank) = 0.33). However, the lengths of hospital stay and in coronary care unit were longer in patients in the Low-zinc group than in those in the High-zinc group (15.6 ± 9.2 days vs. 11.9 ± 2.9 days, P = 0.06; 3.9 ± 2.8 days vs. 2.3 ± 0.8 days, P = 0.01). Multivariate regression analysis showed that low serum zinc concentration was associated with the use of cardiac or respiratory assist devices (adjusted odds ratio, 17.79; 95% CI 1.123 to 1216.5; P = 0.04).

**Conclusions:**

Although there was no significance difference in mortality in Low-zinc and High-zinc groups, low serum zinc concentration was associated with longer stay in the coronary care unit, and was one of the independent predictors for the use of cardiac or respiratory assist devices.

## Introduction

Acute myocardial infarction (AMI) is a major cause of death worldwide. Current guidelines recommend revascularization of infarct-related artery in patient with AMI and it minimizes myocardial necrosis. Cardiomyocytes death in the acute phase of AMI involves not only necrotic change but also programmed cell death (apoptosis) [1]. Pathological studies demonstrated that apoptosis of cardiomyocytes occurred due to hypoxia, myocardial infarction, reperfusion, and/or heart failure [2–4].

Zinc is one of the important components that have effects on metabolic pathways such as the synthesis of nucleic acids and proteins [5]. Zinc deficiency leads to immune dysfunction, impairment of growth, and increased cardiovascular death in patients with diabetes [6, 7]. According to previous study, zinc ions prevent the apoptosis of cells in vivo and vitro [8].

About half a century ago, Wacker et al. and Lindeman et al. showed that the decrement of serum zinc concentration occurred in patients with AMI [9, 10]. More recently Lang et al. demonstrated that acute organ injury rapidly decreases serum zinc concentration [11]. In the clinical setting, Low et al. reported that serum zinc concentration was highly associated with prognosis of AMI [5]. However, in the primary percutaneous coronary intervention (PCI) era, there are no reports regarding the association between serum zinc concentration and prognosis of AMI. Thus, we assessed the relationships between serum zinc concentration, other biomarkers, complications, and prognosis in AMI patients who underwent primary PCI.

## Materials and methods

### Patient population

Between March 2015 and September 2016, we conducted a single-center open-label prospective observational study. We enrolled AMI patients within 24 hours of symptom onset who underwent primary PCI in Showa University Northern Yokohama Hospital. Patients 20 years of age and older were eligible after successful primary PCI if they provided written informed consent. The study protocol was approved by the Institutional Review Board of Showa University Northern Yokohama Hospital, and complied with the Declaration of Helsinki. Myocardial infarction was defined as symptoms of cardiac ischemia and a troponin level above the 99th centile. The definition required new electrocardiographic evidence of ST-segment elevation or left bundle branch block, or angiographic evidence of coronary artery occlusion [12, 13].

The strategies of PCI were performed by the operator’s decision. We excluded patients with hepatic cirrhosis, inflammatory bowel disease, chronic pancreatitis, after enterectomy or pancreaticoduodenectomy, systemic inflammatory disease, sickle cell anemia, or hemodialysis.

Blood samples were taken on admission, every six hours until the peak creatine kinase (CK) was determined, and then every day for at least three days. Serum zinc concentration was obtained within 24 hours after primary PCI and at discharge. Clinical data including history, age, heart rate, systolic blood pressure, diastolic blood pressure, medication, door to balloon time, and laboratory data were collected. The rate of the use of cardiac or respiratory assist devices such as a temporary pacemaker, intra-aortic balloon pump, invasive or non-invasive positive pressure ventilation, or venoarterial extracorporeal membrane oxygenation was also assessed. Follow-up coronary angiography was performed approximately eight months after primary PCI. Clinical follow-up was performed by clinical visits or telephone calls to patients or their relatives. We divided patients into two groups (High-zinc group and Low-zinc group) according to the median serum zinc concentration of 53.5 mg/dL. The primary endpoint was in-hospital death. Secondary endpoints were the rates of the use of cardiac or respiratory assist devices, the lengths of hospital/coronary care unit (CCU) stay, and major cardiovascular events. Major cardiovascular events were defined as the composite of cardiovascular death, myocardial infarction, hospitalization for unstable angina, or coronary revascularization. This trial was registered with the University Hospital Medical Information Network in Japan, UMIN000018844.

### Statistical analysis

Data were analyzed using JMP 11 (SAS Institute, Inc., Cary, NC, USA). Continuous variables were reported as mean ± standard deviation unless otherwise stated. The Low-zinc group and the High-zinc group were compared by unpaired t-test or Wilcoxon rank sum test, as appropriate. Categorical variables were presented as percentages and compared using chi-square test or Fisher’s exact test, as appropriate. Cumulative survival rates were calculated using Kaplan-Meier analysis, and survival curves were compared using the log-rank test. Univariate and multivariate logistic regression analysis were used to estimate the odds ratios and 95% confidence intervals (CI) for the association between serum zinc concentration and the rate of the use of cardiac or respiratory assist devices. Variables with p value being <0.10 were entered into the multivariate logistic regression analysis. We forced age and sex into the model. Variables usually reported in the literature to be associated with prognosis were also forced into the model. We used multivariate logistic regression models with two or three variables since the sample size was small. We also performed linear regression analyses of the relationship between the length of stay in the CCU and variables. Variables with p value <0.10 (door to balloon time, zinc concentration, creatinine level, and blood urea nitrogen) were entered into the model. We forced age and sex into the model. Variance inflation factor was checked to collinearity before variables entered into the model. A two-sided p value being <0.05 was considered significant.

## Results and discussion

### Results

We enrolled 51 consecutive patients with AMI between March 2015 and September 2016. There was only one patient who declined primary PCI. After exclusion of this case, all 50 patients had successful primary PCI and were eligible for the study. The patient characteristics are shown in Table 1.

**Table 1:** Patients characteristics.

	ALL	High Zinc	Low Zinc	P Value
	N = 50	N = 25	N = 25	
Age, years	66.2±11.8	65.3±11.3	67.0±12.4	0.60
Male gender, n (%)	41 (82.0)	21 (84.0)	20 (80.0)	0.71
ST-segment elevation MI, n (%)	42 (84.0)	18 (72.0)	24 (96.0)	0.02
Heart rate, bpm	79.2±21.2	73.0±17.1	85.5±23.3	0.04
Systolic blood pressure, mmHg	144.5±36.0	147.9±39.4	141.1±32.8	0.51
Diastolic blood pressure, mmHg	90.1±26.1	90.7±25.5	89.4±27.1	0.86
Tube feeding before admission, n (%)	0 (0.0)	0 (0.0)	0 (0.0)	-
Anorexia, n (%)	0 (0.0)	0 (0.0)	0 (0.0)	-
Fasting over 24 hours before admission, n (%)	0 (0.0)	0 (0.0)	0 (0.0)	-
Vegetarian, n (%)	1 (2.0)	0 (0.0)	1 (4.0)	0.24
Comorbidities				
Hypertention, n (%)	26 (52.0)	14 (56.0)	12 (48.0)	0.57
Diabetes, n (%)	18 (36.0)	11 (44.0)	7 (28.0)	0.24
Hyperlipidemia, n (%)	24 (48.0)	15 (60.0)	9 (36.0)	0.09
Smoking, n (%)	33 (66.0)	17 (68.0)	16 (64.0)	0.77
Family history, n (%)	10 (20.0)	7 (28.0)	3 (12.0)	0.15
Previous MI, n (%)	2 (4.0)	1 (4.0)	1 (4.0)	1
Door to balloon time, min	135.3±188.0	122.4±88.7	148.1±252.8	0.64
LVEF (%)	54.2±12.5	56.3±11.9	51.9±13.1	0.24
KILLIP >2, n (%)	5 (10.0)	2 (8.0)	3 (12.0)	0.64
Laboratory data on admission				
Hemoglobin, g/dL	14.8 ± 1.6	15.2 ± 1.2	14.5±1.9	0.12
BUN, mg/dL	16.8 ± 5.0	15.3 ± 2.8	18.4±6.2	0.03
Creatinine, mg/dL	0.82 ± 0.20	0.78 ± 0.16	0.86±0.24	0.18
eGFR, mL/min/1.73m2	72.7 ± 18.2	76.7 ± 17.0	68.6±18.8	0.11
HDL-cholesterol, mg/dL	44.6 ± 12.1	43.5 ± 11.4	45.6±12.9	0.53
LDL-cholesterol, mg/dL	126.6 ± 35.6	135.8 ± 37.5	117.8±31.9	0.08
Glucose, mg/dL	178.6 ± 81.2	170.6 ± 60.9	186.7±98.2	0.49
Hemoglobin A1c, %	6.6 ± 1.5	6.5 ± 1.2	6.7±1.8	0.61
CK, IU/L	508.2 ± 733.5	362.0 ± 357.1	654.4±962.5	0.16
CK-MB, IU/L	49.6 ± 85.7	34.1 ± 30.5	65.0±116.5	0.20
Peak CK, IU/L	2569.3 ± 2417.3	2824.4 ± 2717.9	2314.2±2099.5	0.46
Sodium, mEq/L	138.8 ± 2.5	139.0 ± 2.2	138.7±2.8	0.74
Potassium, mEq/L	4.1 ± 0.5	3.9 ± 0.4	4.2±0.6	0.10
Zinc within 24 hours	55.9 ± 12.5	65.5 ± 9.5	46.4±6.3	<0.001
Zinc at discharge	73.9 ± 12.9 *	78.5 ± 13.1 *	69.1±11.0 *	0.01
Medication at discharge				
Aspirin	48 (100)	24 (100.0)	24 (100.0)	1
ACEI/ARB	44 (91.7)	21 (87.5)	23 (95.8)	0.29
Beta blocker	41 (85.4)	20 (83.3)	21 (87.5)	0.68
Statin	45 (93.8)	24 (100.0)	21 (87.5)	0.07
Aldosterone antagonist	8 (16.7)	3 (12.5)	5 (20.8)	0.44

“*” means Paired t-test for between group P value <0.001

Data are expressed as numbers and/or percentages or means and standard deviation.

Abbreviations: ACEI; angiotensin converting enzyme inhibitor; ARB, angiotensin 2 receptor blocker; BUN, blood urea nitrogen; CK, creatine kinase; GFR, glomerular filtration rate; LVEF, left ventricular ejection fraction; STEMI, ST elevation myocardial infarction

The mean age of patients was 66.2 ± 11.8 years and 82% of patients were male. Patients in the Low-zinc group had ST-segment elevation MI more frequently and had higher heart rate than those in the High-zinc group (96.0% vs. 72.0%, P = 0.02; 85.5 ± 23.3 bpm vs. 73.0 ± 17.1 bpm, P = 0.04). Comorbidities were similar in the both groups. Serum zinc concentration significantly increased at discharge compared to that within 24 hours after primary PCI in the both groups (from 46.4 ± 6.3 mg/dL to 69.1 ± 11.0 mg/dL, P < 0.001; from 65.5 ± 9.5 mg/dL to 78.5 ± 13.1 mg/dL, P = 0.02). Peak CK level was not significantly different in the both groups. In-hospital course and post-discharge outcomes are shown in Table 2.

**Table 2:** In-hospital and post discharge outcomes.

	ALL	High Zinc	Low Zinc	P Value
	n = 50	n = 25	n = 25	
In-hospital death, n (%)	2 (4.0)	1 (4.0)	1 (4.0)	1
Use of cardiac or respiratory assist devices, n (%)	15 (30.0)	4 (16.0)	11 (44.0)	0.03
Length of hospital stay, day	13.8±7.0	11.9±2.9	15.6±9.2	0.06
Length of stay in coronary care unit, day	3.1±2.2	2.3±0.8	3.9±2.8	0.01
Major cardiovascular events, n (%)	9 (18.0)	3 (12.0)	6 (24.0)	0.27
All-cause death, n (%)	4 (8.0)	1 (4.0)	3 (12.0)	0.29
Cardiovascular death, n (%)	2 (4.0)	1 (4.0)	1 (4.0)	1
Myocardial infarction, n (%)	0 (0.0)	0 (0.0)	0 (0.0)	-
Target vessel revascularization, n (%)	3 (6.0)	1 (4.0)	2 (8.0)	0.55
Hospitalization of unstable angina, n (%)	2 (4.0)	1 (4.0)	1 (4.0)	1

There was no significant difference in in-hospital death between the two groups (4.0% vs. 4.0%, P = 1.0). The prevalence of ventricular arrhythmia was also similar in the both groups (Table 2). The rate of the use of cardiac or respiratory assist devices was significantly higher in patients in the Low-zinc group than that in the High-zinc group (48.0% vs. 16.0%, P = 0.01). The length of hospital stay and that in the CCU were longer in patients in the Low-zinc group than in those in the High-zinc group (15.6 ± 9.2 days vs. 11.9 ± 2.9 days, P = 0.06; 3.9 ± 2.8 days vs. 2.3 ± 0.8 days, P = 0.01, respectively). The multivariate logistic regression analyses of the use of cardiac or respiratory assist devices are shown in Table 3.

**Table 3:** Logistic regression analysis of low serum zinc concentration for the use of cardiac or respiratory assist devices.

	Odds ratio	95% CI		P Value
Unadjusted	4.125	1.157	17.36	0.03
Adjusted (age, sex)	4.722	1.157	24.84	0.03
Adjusted (age, sex, hemoglobin)	4.345	1.014	2370	0.048
Adjusted (age, sex, systolic blood pressure)	6.283	1.236	47.14	0.03
Adjusted (age, sex, heart rate)	8.757	1.746	65.37	0.007
Adjusted (age, sex, peak CK)	19.99	2.611	357.95	0.002
Adjusted (age, sex, KILLIP)	6.727	1.267	59.54	0.02
Adjusted (age, sex, systolic blood pressure, peak CK, KILLIP)	17.79	1.123	1216.5	0.04

Abbreviations: CK, creatine kinase

Low zinc concentration was one of the independent predictors for the use of those devices (adjusted odds ratio, 17.79; 95% CI 1.123 to 1216.5; P = 0.04). The multivariate linear regression analyses showed that low serum zinc concentration was one of the independent predictors for long length of stay in the CCU except for the model with age, sex, and blood urea nitrogen (β = -0.612; 95% CI -1.136 to -0.088; P = 0.02) (Table 4).

**Table 4:** Linear regression analysis of zinc concentration for the length of stay in the CCU.

	Beta coefficients	Standardized coefficients	95% CI		P value
Unadjusted	-0.052	-0.283	-1.050	0.0002	0.05
Adjusted (age, sex)	-0.725	-0.334	-1.336	-0.115	0.02
Adjusted (age, sex, Cre)	-0.622	-0.287	-1.241	-0.004	0.049
Adjusted (age, sex, BUN)	-0.520	-0.239	-1.137	0.096	0.10
Adjusted (age, sex, DTB)	-0.668	-0.308	-1.170	-0.167	0.01
Adjusted (age, sex, heart rate)	-0.572	-0.263	-1.191	0.047	0.07
Adjusted (age, sex, KILLIP)	-0.696	-0.320	-1.304	-0.088	0.03
Adjusted (age, sex, DTB, BUN, Cre)	-0.612	-0.282	-1.136	-0.088	0.02

Abbreviations: BUN, blood urea nitrogen; Cre, creatinine; DTB, door to balloon time

**Fig 1:**
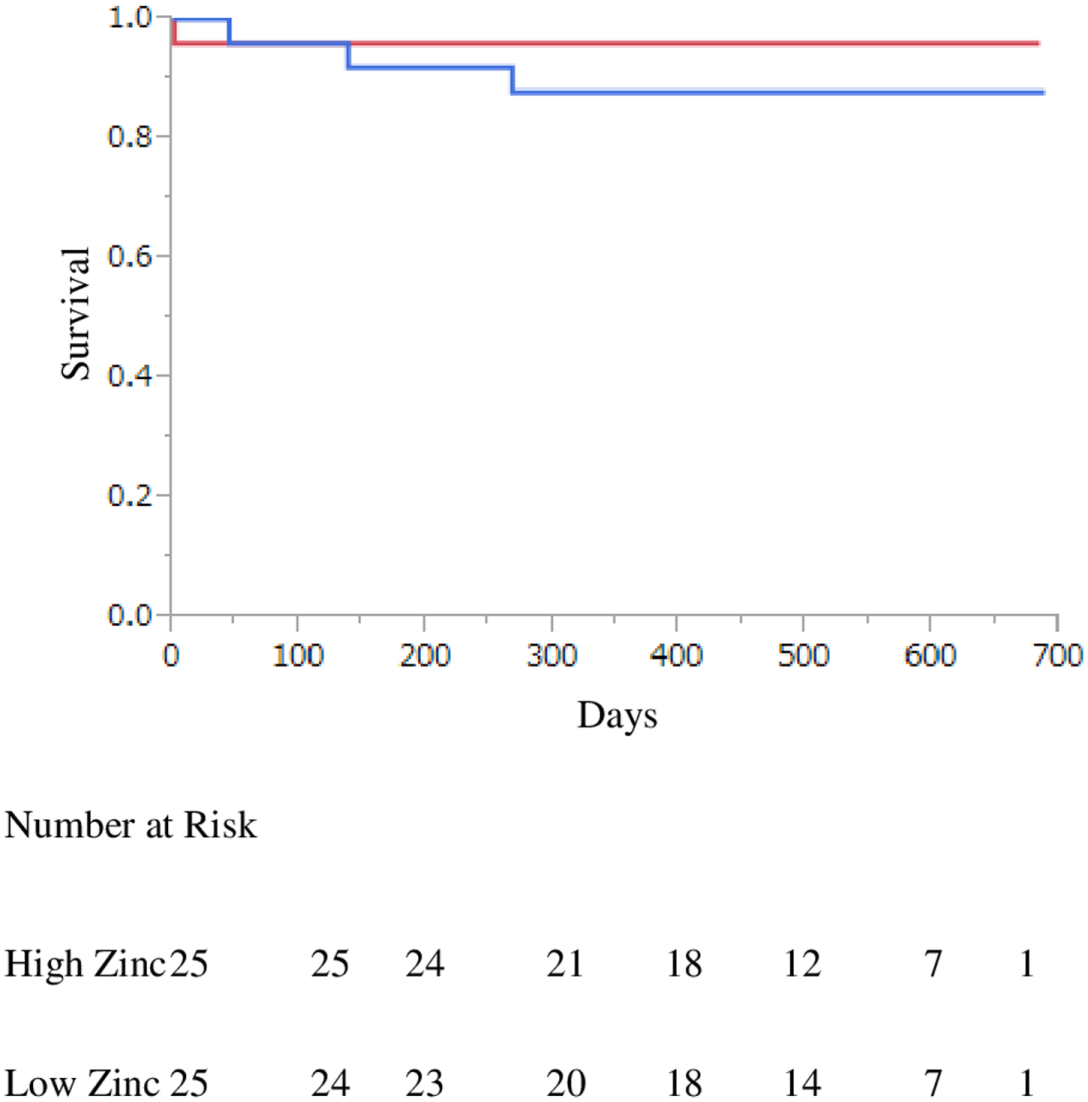
Kaplan-Meier analysis of the freedom from all-cause death and the major cardiovascular events. A is a Kaplan-Meier survival curve for the patients in Low-zinc group (blue line) and in High-zinc group (red line). B is a Kaplan-Meier major cardiovascular event free survival curve for the patients in Low-zinc group (blue line) and in High-zinc group (red line).

Clinical follow-up was completed in all patients. The angiographic follow-up rate was 72%. During a median follow-up period of 501 days (interquartile range 391–612 days), there were no significant differences in all-cause mortality and major cardiovascular events in the both groups by Kaplan-Meier analysis (P log-rank = 0.33, P log-rank = 0.29) (Fig 1).

### Discussion

In the present study, 1) serum zinc concentration was not associated with in-hospital and all-cause mortality in patients with AMI who underwent primary PCI. However, 2) low serum zinc concentration was associated with the higher rate of the use of cardiac or respiratory assist devices and the longer length of stay in the CCU. In addition, 3) serum zinc concentration increased promptly during hospitalization.

To our knowledge, there were no reports regarding the relationship between serum zinc concentration and mortality in patients with AMI in the era of primary PCI. According to a study in diabetic patients, low serum zinc concentration was associated with cardiovascular death [7]. However, our finding did not show the association between serum zinc concentration and all-cause mortality. These results might have been derived from low statistical power of the present study due to the limited number of cases.

Our findings were consistent with previous studies regarding the association between serum zinc concentration and the length of stay in hospital [1, 2]. In addition, low serum zinc concentration was associated with high rate of use of cardiac or respiratory assist devices. Taken together, it is presumed that low serum zinc concentration possibly reflected the severity of MI (longer hospital stay or more frequent use of cardiac or respiratory assist devices). In addition, patients with Low-zinc groups had a higher BUN level than High-zinc group. The reduced cardiac output and renal congestion secondary to AMI might increase the reabsorption of urea in proximal tubes [14]. This mechanism might be the reason that BUN increased more in the Low-zinc group in the present study.

Based on the normal range of serum zinc concentration (from 59 μg/dL to 135 μg/dL by BML (Tokyo, Japan)), we suppose that serum zinc concentration rapidly decreased in the acute phase of AMI and recovered during hospital stay. Some explanations of this serum zinc concentration reduction are as follows. Serum zinc concentration rapidly decreases at the beginning of the acute phase response because zinc is redistributed to the intracellular space in patients with critical illness [15]. Inflammatory cytokines have effects on both up- and down-regulation of the expression of the zinc transporters such as ZnT and Zip. An increased demand for Zn-dependent proteins in the inflammatory status alters zinc transporter expression profile and this leads to elevated intracellular zinc concentration [11]. From these mechanisms of zinc redistribution in critical illness, it is speculated that in patients with AMI, zinc may move into the cellular compartment and serum zinc concentration may decrease.

Some previous reports showed that increased inflammatory cytokines derived from the organ injury or heart failure secondary to AMI are related with reduction of serum zinc concentration [15, 16]. These phenomena may cause initial reduction of serum zinc concentration and its subsequent recovery within two weeks after the onset of AMI.

Does zinc supplementation become a therapeutic option in patients with AMI? Zinc is one of the stabilizers of lipids and proteins and protects cellular membranes from oxidative damage [17]. The experimental study reported that apoptosis may be a specific feature of reperfusion injury in cardiac myocytes [3]. Hofstra et al. demonstrated that the apoptosis of myocytes was frequently detected by the increased uptake of annexin-V in AMI patients who underwent primary PCI within 6 hours from onset [4]. Thus serum zinc concentration might decrease after primary PCI, because apoptosis persists as long as 10 days after AMI [1]. It has been reported that apoptosis was prevented by zinc administration in animal models due to the inhibition of caspase-3, which is one of the mediators of apoptosis [17]. In a study with healthy individuals, it was demonstrated that zinc supplementation decreased the concentrations of high-sensitivity C-reactive protein and IL-6 [18]. These suggest that zinc supplement in AMI may be effective in decreasing infarct size or in suppressing inflammation.

There was no randomized trial, to our knowledge, that investigated the association between zinc supplement and renal function. However, Bolignano et al. showed that antioxidant agents including zinc reduced albuminuria in patients with diabetic kidney disease [19]. In an animal study, zinc reduced apoptosis and inflammation in kidney after ischemia and reperfusion [20]. Renal impairment was associated with poor prognosis in patient with AMI and heart failure [21, 22]. Renal congestion and low perfusion of kidney due to heart failure induces renal impairment and subsequent inflammation of the kidney [14]. Therefore zinc supplement may also protect the kidney from inflammation and improve outcomes in patients with AMI.

### Limitations

The present study was a small-sized single-center prospective study. The number of events was few. Our study population might not represent the entire population with AMI. Analyses using multivariate Cox proportional hazards model were performed, but unknown confounders might have impacted the analyses. In hospital treatment including PCI procedure was determined by treating cardiologist’s discretion. The nutritional status before admission was not obtained. We used peak CK as the only indicator of myocardial infarct size, since cardiac MRI was not used. The present study might have been underpowered to detect an association between serum zinc concentration and prognosis.

## Conclusions

Although there was no significance difference in mortality in Low-zinc and High-zinc groups, low serum zinc concentration was associated with longer stay in the coronary care unit, and was one of the independent predictors for the use of cardiac or respiratory assist devices.

## Supporting information

S1 Fig The flow chart of the present study. The flow chart of the present study is shown according to CONSORT 2010.
(DOC)

S2 Fig Protocol. Protcol is translated from the original language to English briefly.
(DOCX)

S3 Fig Original protocol. Original protocol is written in the original language.
(DOCX)

## Abbreviations

ACEI: angiotensin converting enzyme inhibitor
AMI: acute myocardial infarction
ARB: angiotensin 2 receptor blocker
BUN: blood urea nitrogen
CCU: coronary care unit
CK: creatine kinase
Cre: creatinine
DTB: door to balloon time
GFR: glomerular filtration rate
LVEF: left ventricular ejection fraction
PCI: percutaneous coronary intervention
