# Supplementary material for: Serum zinc concentration in patients with acute myocardial infarction in percutaneous coronary intervention era
Source: PLoS One. 2018 Aug 30;13(8):e0203074. doi: 10.1371/journal.pone.0203074 (PMC6117045; doi:10.1371/journal.pone.0203074)
Supplement: S1 Fig — The flow chart of the present study is shown according to CONSORT 2010. (DOC) [file pone.0203074.s001.doc]

**
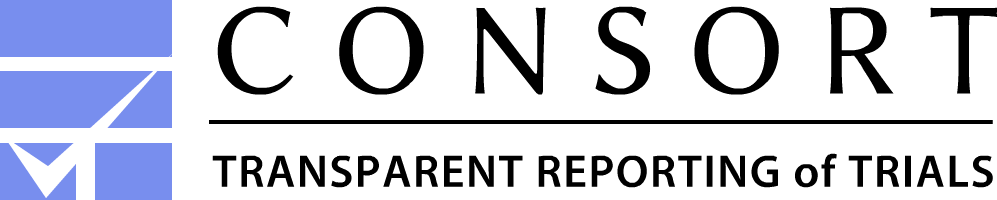
**

**CONSORT 2010 Flow Diagram**

**Allocation**

**Analysis**

**Follow-Up**

**Enrollment**

Assessed for eligibility (n=51)

Excluded (n=1)

♦  Not meeting inclusion criteria (n=0)

♦  Declined to participate (n=1)

♦  Other reasons (n=0)

Analysed (n=25)
♦ Excluded from analysis (give reasons) (n=0)

Lost to follow-up (give reasons) (n=0)

High-Zinc group (n=25)

Serum zinc concentration >53.5 mg/dL

Lost to follow-up (give reasons) (n=0)

Low-Zinc group (n=25)

Serum zinc concentration ≤53.5 mg/dL

Analysed (n=25)
♦ Excluded from analysis (give reasons) (n=0)

Divided by mean serum zinc concentration (n=50)
