# Supplementary material for: Serum zinc concentration in patients with acute myocardial infarction in percutaneous coronary intervention era
Source: PLoS One. 2018 Aug 30;13(8):e0203074. doi: 10.1371/journal.pone.0203074 (PMC6117045; doi:10.1371/journal.pone.0203074)
Supplement: S2 Fig — Protcol is translated from the original language to English briefly. (DOCX) [file pone.0203074.s002.docx]

**Methods**

**Institution:** Showa University Northern Yokohama Hospital

**Primary endpoint：**Association between serum zinc concentration, in-hospital mortality, 1 year all-cause mortality, and major cardiovascular events.

**Secondary endpoint：**Association between severity of myocardial infarction such as laboratory data, LVEF, NYHA class, and length of CCU/hospital stay.

**Safety assessment:** None. This study is observational study.

**Data collection:**

Blood samples were taken on admission, every six hours until the peak creatine kinase (CK) was determined, and then every day for at least three days. Serum zinc concentration was obtained within 24 hours after primary PCI and at discharge. Clinical data including history, age, heart rate, systolic blood pressure, diastolic blood pressure, medication, door to balloon time, and laboratory data were collected. The rate of the use of cardiac or respiratory assist devices such as a temporary pacemaker, intra-aortic balloon pump, invasive or non-invasive positive pressure ventilation, or venoarterial extracorporeal membrane oxygenation was also assessed. Follow-up coronary angiography was performed approximately eight months after primary PCI. Clinical follow-up was performed by clinical visits or telephone calls to patients or their relatives.

**Termination of study**

1）If patients need to quit the study

2）If we cannot able to follow participants, because they move abroad.

3）If after enrollment, we find that participants meet exclusion criteria.

4）If treatment physician make the decision to stop the study because of severe adverse event associated with myocardial infarction.

5）Protocol deviations

8）If some specific reason that make us not to continue the study occur.

**Analysis**

Data were analyzed using JMP 11 (SAS Institute, Inc., Cary, NC, USA). Continuous variables were reported as mean ± standard deviation unless otherwise stated. The Low-zinc group and the High-zinc group were compared by unpaired t-test or Wilcoxon rank sum test, as appropriate. Categorical variables were presented as percentages and compared using chi-square test or Fisher’s exact test, as appropriate. Cumulative survival rates were calculated using Kaplan-Meier analysis, and survival curves were compared using the log-rank test. Univariate and multivariate logistic regression analysis were used to estimate the odds ratios and 95% confidence intervals (CI) for the association between serum zinc concentration and the rate of the use of cardiac or respiratory assist devices. Variables with p value being <0.10 were entered into the multivariate logistic regression analysis. We forced age and sex into the model. Variables usually reported in the literature to be associated with prognosis were also forced into the model. We used multivariate logistic regression models with two or three variables since the sample size was small. We also performed linear regression analyses of the relationship between the length of stay in the CCU and variables. Variables with p value <0.10 (door to balloon time, zinc concentration, creatinine level, and blood urea nitrogen) were entered into the model. We forced age and sex into the model. Variance inflation factor was checked to collinearity before variables entered into the model. A two-sided p value being <0.05 was considered significant.

**Duration of study**

Duration is from after approval by the Institutional Review Board of Showa University Northern Yokohama Hospital to December 2017.

**Criteria**

**Including criteria**

a) AMI patients within 24 hours of symptom onset who underwent primary PCI in Showa University Northern Yokohama Hospital.

b) Patients 20 years of age and older

c) Patients provided written informed consent.

**Exclusion criteria**

Patients with hepatic cirrhosis, inflammatory bowel disease, chronic

pancreatitis, after enterectomy or pancreaticoduodenectomy, systemic

inflammatory disease, sickle cell anemia, or other reason that we decide to exclude the patients such as hemodialysis.

**Patient number**

We did not perform power analysis, because there are no data of the association between serum zinc concentration and mortality. In our hospital, about 50 cases of acute myocardial infarction cases are treated per year.

**Ethics**

The study protocol was approved by the Institutional Review Board of Showa University Northern Yokohama Hospital, and complied with the Declaration of Helsinki.
