# Supplementary material for: Serum zinc concentration in patients with acute myocardial infarction in percutaneous coronary intervention era
Source: PLoS One. 2018 Aug 30;13(8):e0203074. doi: 10.1371/journal.pone.0203074 (PMC6117045; doi:10.1371/journal.pone.0203074)
Supplement: S3 Fig — Original protocol is written in the original language. (DOCX) [file pone.0203074.s003.docx]

**研究実施計画書**

**1 研究の名称**

血清亜鉛濃度と心筋梗塞重症度・予後との関連の前向き観察研究

**2　研究の目的及び意義(当該研究の臨床的意義を明記)**

| 本研究は、急性心筋梗塞患者を対象とし、入院時、入院中、退院時、外来受診時の血清亜鉛濃度を測定し、心筋梗塞入院日数、BNPを含む血液生化学検査、1年予後を含めた中長期的予後との関連を検証することを目的とした前向き観察研究である。 |
| --- |

**3 研究の方法及び期間**

**3-1）実施施設**

試験は昭和大学横浜市北部病院心臓血管カテーテル室の入院病棟並びに外来で実施し、急性心筋梗塞患者の通常の診療内の血液検査で得られた結果を用いる。

**3-2）評価項目**

**1）主要評価項目（Primary endpoint）：**

血清亜鉛濃度と入院中死亡，心血管イベント・再入院・死亡などの予後との相関

**2）副次評価項目（Secondary endpoint）**

心機能，腎機能，肝機能，NYHA class, KILLIP，入院期間など重症度との相関

**3）安全性評価項目**

**3-3）試験薬概要（用法・用量、投与期間、予測される副作用）**

本研究は介入研究ではなく観察研究であり、試験薬はない。

**3-4）試験薬の割付**

本研究は介入研究ではなく観察研究であり、試験薬はない。

**3-5）試料（情報）と入手方法**

試験開始前

被験者の組み入れ時（入院日）において、診療録から下記の情報を収集する。

被験者背景：年齢（生年月日）、性別、身長・体重、血圧、経皮的動脈血酸素飽和度

被験者の同意：同意者、同意取得年月日

原疾患：初回発病年齢、発症年月日（診断日）、罹患期間、重症度

原疾患の治療歴：現在までに使用した治療薬の名称及びその中止理由（当てはまる場合）

合併症：（同意取得時に罹患中の疾患）有・無、疾患名

既往歴：（同意取得時までに治癒した疾患）

現在の併用薬：薬剤名、一日投与量、投与経路、投与理由、投与期間など）

また、臨床検査ならびに・・・・に関する下記のデータをあわせて収集する。

血液学的検査：赤血球数、ヘモグロビン量、ヘマトクリット値、白血球数、白血球分画［St, Seg, Lym, Mo, Eo, Ba］、血小板数

血液生化学検査：TP, Alb, AST (GOT), ALT (GPT), Al-P, LDH, γ-GTP, 総ビリルビン, BUN, クレアチニン, eGFR，総コレステロール, LDL-コレステロール, HDL-コレステロール，トリグリセリド，BNP，CRP，血清亜鉛，Na，K，Cl，CK，CK-MB

血清学的検査：HBs抗原、梅毒検査、HCV抗体

尿検査）：蛋白、糖、ウロビリノーゲン、ケトン体

特殊検査：心エコー図，心電図

血液生化学検査：静脈或いは動脈より最低1回6mLの血液を採取する。これは通常の診療で必要な生化学検査資料の量である。また採血回数も通常の診療の範囲で行う回数である。本研究はこの検体量や採血回数に追加し更なる検体を必要としない。このように本試験は被験者に対し軽微な侵襲以上を与えることはなく、概要のとおり観察研究であり介入研究ではない。

血清亜鉛測定時

入院時(第1病日)，第2,3,5,7病日, 退院時, 退院後初回外来受診時

血液学的検査：赤血球数、ヘモグロビン量、ヘマトクリット値、白血球数、白血球分画［St, Seg, Lym, Mo, Eo, Ba］、血小板数

血液生化学検査：TP, Alb, AST (GOT), ALT (GPT), Al-P, LDH, γ-GTP, 総ビリルビン, BUN, クレアチニン, eGFR，総コレステロール, LDL-コレステロール, HDL-コレステロール，トリグリセリド，BNP，CRP，血清亜鉛，Na，K，Cl，CK，CK-MB

なお、試験担当医師による被験者の安全性確認（バイタルサインの確認、有害事象の聴取）は通常診療における経過観察に準じて試験期間中を通して行なう。

| 評価項目 | **試験**  **開始日** | **第2病日** | **第3病日** | **第5病日** | **第7病日** | **退院時** | **外来受診時** |
| --- | --- | --- | --- | --- | --- | --- | --- |
| 同意取得 | ● |  |  |  |  |  |  |
| 背景調査 | ● |  |  |  |  |  |  |
| 適格性確認 | ● |  |  |  |  |  |  |
| 血液・尿検査 | ● | ● | ● | ● | ● | ● | ● |
| 特殊検査など | ● |  |  |  |  | ● |  |
| 副作用の調査 |  | | | | | | |

**3-6）試験中止基準**

試験担当医師は、試験期間中に下記に該当する被験者が発生した場合には、当該被験者に対する試験を中止する。また試験の中止又は中断を決定した時は、被験者に対する適切な対応をするとともに、速やかに機関の長にその理由とともに文書で報告する。

1）被験者（又は代諾者）より中止の申し入れがあった場合

2）被験者の都合により試験が中断された場合（転居、転医・転院、多忙、追跡不能等）

3）試験開始後、被験者が対象症例ではないことが判明した場合

4）偶発的な事故が発生した場合

5）有害事象が発現し（原疾患の増悪、合併症の増悪又は偶発症を含む）、試験担当医師が中止すべきと判断した場合

6）効果不十分又は症状悪化のため、試験の継続が困難となった場合

7）本試験実施計画書から重大な逸脱があり評価不能と判断される場合

8）被験者が試験担当医師の指示どおり服薬していないことが判明した場合

9）その他、試験担当医師が試験の継続を困難と判断し中止が妥当と判断した場合

**3-7）解析方法**

Per Protocol Set（PPS）：研究計画書をすべて遵守した症例のみを解析の対象とする。

本研究は、今後規模を大きく実施する検証試験の症例数、解析法の設定を考慮する際の情報の収集を目的としているため、詳細な解析手法は設定していない。本試験の評価項目として設定した種々の検査・観察項目のデータのカテゴリーに従い、適切な統計解析を、統計ソフトを用いて解析比較する。

**3-8）研究期間**

昭和大学北部病院倫理委員会承認後から2017年12月31日まで

**4 研究対象者の選定方針**

**4-1）選択基準**

昭和大学横浜市北部病院心臓血管カテーテル室へ入院した心筋梗塞患者を対象し、次の全ての条件を満たすものを本試験の対象とする

　　　　(1) 20歳以上の男女

　　　　(2) 同意のとれた患者

**4-2）除外基準**

(1)肝硬変の患者

(2)維持血液透析の患者

(3)炎症性腸疾患の患者

(4)慢性膵炎の患者

(5)膵広範切除術後・小腸切除術後の患者

(6)膠原病・リウマチ疾患の患者

(7)鎌状赤血球症の患者

(8)上記以外で、研究担当医が不適当と判断した患者

**4-3）目標症例数**

目標症例数は約50名程度

**4-4）設定根拠**

これまでに、血清亜鉛濃度と急性心筋梗塞患者の合併症、重症度との報告が行われているが、30年以上前の報告であり、現在のようなprimary PCI時代の急性心筋梗塞症例を対象とした検証はなされていない。そのため本試験においても、これまでの報告に基づき、有意水準ならびに検出力を考慮する、統計学的検討による症例数の設定は行っていない。

本試験は、評価項目として心血管イベント・死亡を設定して実施するが、それらの結果を踏まえ、今後さらに規模を拡大した介入試験の実施を予定している。そのため本試験は、パイロット試験の意味合いが強い探索的試験としての位置づけであるため、当施設にて行いうる最大人数、ならびに過去の研究を参考として50人と設定した。

**5 研究の科学的合理性の根拠**

亜鉛は生体の必須微量元素のひとつである。亜鉛欠乏が味覚障害や腸性肢端皮膚炎を引き起こすことが広く知られている。1950年代～80年代に急性心筋梗塞発症後24時間以内に血清亜鉛が著明に低下することや心筋梗塞後の予後因子であることを示唆する研究が数例あるものの[1-2]、以降循環器疾患との関連はさほど検証されていない。また亜鉛補充がその予後を改善するかも検証されていない。現在は急性心筋梗塞に対してはprimary PCIによる血行再建がゴールデンスタンダードの治療として確立したPCI時代においては、心筋梗塞患者に対して亜鉛が果たす役割は検証されていないと言って過言ではない。本研究により血清亜鉛が心筋梗塞患者のPCI後の再狭窄や予後予測因子となりうるかを検証できうる。また亜鉛補充が心筋梗塞に対する新たな補助的治療となりうるか、本研究結果によっては更なる研究を検討する。

[1] Wacker WEC, et al : Metalloenzymes and myocardial infarction. Ⅱ. Malic and lactic dehydrogenase activities and zinc concentrations in serum. N Engl J Med 255: 449, 1956

[2] Singh R, et al : Serum zinc in myocardial infarction. Diagnostic and prognostic significance. Angiology 34: 215, 1983

**6 インフォームド・コンセントを受ける手続等（インフォームド・コンセントを受ける場合には、同規定による説明及び同意に関する事項を含む。）**

1）事前に昭和大学横浜市北部病院の臨床試験審査委員会で承認の得られた説明文書･同意文書を研究対象者に渡し、文書及び口頭による十分な説明を行い、研究対象者の自由意思による同意を文書で得る。

2）研究対象者の同意に影響を及ぼすと考えられる有効性や安全性等の情報が得られた時や、研究対象者の同意に影響を及ぼすような実施計画等の変更が行われる時は、速やかに研究対象者に情報提供し、試験等に参加するか否かについて研究対象者の意思を予め確認するとともに、事前に臨床試験審査委員会の承認を得て説明文書・同意文書等の改訂を行い、研究対象者の再同意を得る。

3）研究対象者が未成年や被験者の医療上の問題から同意取得が困難な場合には代諾者から同意を得る。

4）説明文書・同意文書には、研究対象者が理解しやすい表現に配慮し作成する。

**7 個人情報等の取扱い（匿名化する場合にはその方法を含む。）**

本試験は、ヘルシンキ宣言に基づく倫理的原則及び人を対象とする医学系研究に関する倫理指針（2014年12月22日厚生労働省）に従い、本試験実施計画書を遵守して実施する。

本研究で取り扱う試料・情報等は、個人情報管理責任者が連結不可能匿名化したうえで、研究・解析に使用する。すなわち連結不可能匿名化を行うため、試料・情報から個人を識別できる情報（氏名、住所、生年月日、電話番号など）を削除し、個人と符号の対応表は作成しない。

**8 研究対象者に生じる負担並びに予測されるリスク及び利益、これらの総合的評価並びに当該負担及びリスクを最小化する対策**

本研究で実施する臨床検査は通常の診療で行なわれる検査及び治療目的の範囲を越えず、それによって患者に大きな不利益が生じることはない。

**9 試料・情報（研究に用いられる情報に係る資料を含む。）の保管及び廃棄の方法**

**9-1）試料の保管及び廃棄の方法**

本研究終了後において、本研究で得られた被験者試料を他の研究において使用することはない。研究終了時には、全ての試料は速やかに破棄する。

**9-2）情報の保管及び破棄の方法**

被験者の本研究終了後に継続する通常医療活動において活用される従来の診療情報については、医師法等の関連法規に従い保管する。本研究の実施のために匿名化され取得した研究関連情報については、研究責任者あるいは分担研究者の所属する施設のコンピューターを用いて移動媒体内に保存するか、外部から切り離されたコンピューターのハードディスク内に保存する。情報を取り扱う研究者は、研究情報を取り扱うコンピューター及び移動媒体をパスワード管理するなどにより、情報の紛失・漏洩等に十分配慮した取扱いの上での保管を行う。

**9-3）情報の保管期間**

研究責任者は、試験終了後、速やかに医療機関の長に試験の終了報告書を提出するとともに、研究等の実施に係わる重要な文書（申請書類の控え、病院長からの通知文書、各種申請書・報告書の控、同意書、症例報告書、その他データの信頼性を保証するのに必要な書類又は記録等）を、研究の中止又は終了後少なくとも5年間、あるいは研究結果発表後3年が経過した日までの間のどちらか遅い期日まで保存する。

なお、通常診療に用いる医療情報の保管・破棄は関連法規（医師法）等の規定に従うこととする。

**10 研究機関の長への報告内容及び方法**

試験薬との因果関係の有無にかかわらず、重篤な有害事象が発現した場合は、試験担当医師は、安全確保を第一優先に迅速かつ適切な処置を講じた後、速やかに昭和大学横浜市北部病院 病院長及び昭和大学横浜市北部病院の臨床試験審査委員会 委員長に報告するとともに、病院長による厚生労働大臣への報告ならびに公表について協力する。

また、研究の実施状況について１年に１回以上「研究終了/経過報告書(研究)」又は「先進医療終了報告書/経過報告書」を用いて研究機関の長に報告する。

**11 研究の資金源等、研究機関の研究に係る利益相反及び個人の収益等、研究者等の研究に係る利益相反に関する状況**

本試験の計画、実施、発表に関して可能性のある利益相反（conflict of interest）はない。利益相反とは、研究成果に影響するような利害関係を指し、金銭及び個人の関係を含む。

本研究は、昭和大学横浜市北部病院心臓血管カテーテル室が計画し実施する自主臨床研究であり本研究に使用する医薬品の製造販売を行っている製薬会社をはじめ、他の団体からの資金的援助に基づいて行われるものではない。

**12 研究に関する情報公開の方法**

**12-1）研究実施計画書の登録**

本臨床研究は、被験者への医療的介入を行なうものではないため、ヘルシンキ宣言ならびに人を対象とする医学系研究に関する倫理指針（2014年12月22日厚生労働省）で求める臨床研究計画の事前登録の要件には該当しない。

**12-2）知的財産権及び研究結果の公表について**

この研究から特許権、また、それを基として経済的利益が生じる可能性があるが、その権利は研究を実施する研究機関や研究者に属し、本試験の被験者がこの権利を持つことはない。また本試験実施計画書に基づいて行われた試験成績は、研究会及び実施医療機関の共有のものとする。成績の公表に関する事項は、研究会により決定する。公表の際には被験者の個人情報を保全する。

**13 研究対象者等及びその関係者からの相談等への対応**

本研究に同意した後でも、疑問や不明な点があった場合は、自由に研究者への質問を受け入れる。研究者がその時点で得られる情報をもとに返答する。また、本研究の計画及び方法についての資料はいつでも閲覧可能とする。

≪連絡先≫

担当者：岡部　俊孝 昭和大学横浜市北部病院　心臓血管カテーテル室　助教(院外)

045-949-7000(内線7727)

**14 代諾者等からインフォームド・コンセントを受ける場合**

本研究では試験への組入れ評価の時点において、被験者の健康上の問題から本人同意を取得できない場合が想定される。その場合においては、必ず代諾者となるべき者より同意を得て、当該被験者となるべき者を治験に参加させるものとする。

**15 インフォームド・アセントを得る場合**

15歳未満を対象としないため該当せず

**16 緊急かつ明白な生命の危機が生じている状況における研究の実施（指針第12の5の規定）**

　　研究対象者に緊急かつ明白な生命の危機が生じている状況で、本研究は通常診療の血液検査・臨床検査以上の侵襲はないため同意が得られ、試験に参加するとなった場合のリスク負担は必要最小限である。

**17 研究対象者等に経済的負担又は謝礼がある場合には、その旨及びその内容**

本試験に伴う試験薬の処方、診察・検査等は通常の保険診療に準じて行うため研究実施による発生する医療費は被験者負担にて行う。

**18 侵襲（軽微な侵襲を除く。）を伴う研究の場合には、重篤な有害事象が発生した際**

**の対応**

本研究は通常診療内の血液検査・臨床検査の域を超えることはなく軽微な侵襲と考えられるため根本的本項目には該当しないと考えられるが、発生した有害事象の治療は、原則として通常の保険診療にて行うものとするが、治療においてはできる限り被験者の負担とならないよう十分に配慮する。なお、発現した有害事象に対して行なった治療は適切に診療録等に記録する。

**19 侵襲を伴う研究の場合には、当該研究によって生じた健康被害に対する補償の有無**

**及びその内容**

　　本試験の実施により研究協力者に健康被害が生じた場合には、研究責任者又は分担研究者は十分な治療等の適切な措置を行うとともに、その原因を究明に努める。医療費などの金銭的補償は行わない。

**20 通常の診療を超える医療行為を伴う研究の場合には、研究対象者への研究実施後に**

**おける医療の提供に関する対応**

通常の診療を超える医療行為を伴わないため該当せず。

**21 研究の実施に伴い、研究対象者の健康、子孫に受け継がれ得る遺伝的特徴等に関す**

**る重要な知見が得られる可能性がある場合には、研究対象者に係る研究結果（偶発**

**的所見を含む。）の取扱い**

本研究において実施した検査・観察項目により、被験者の医療上の問題が偶発的に発見される場合が想定される。その場合には、被験者に対してその旨を十分に説明するとともに、必要に応じて偶発的に発見された医療上の問題点に関する専門医への相談・紹介等を行う。

**22 研究に関する業務の一部を委託する場合には、当該業務内容及び委託先の監督方法**

業務を委託しないため該当せず。

**23 研究対象者から取得された試料・情報について、研究対象者等から同意を受ける時**

**点では特定されない将来の研究のために用いられる可能性又は他の研究機関に提供**

**する可能性がある場合には、その旨と同意を受ける時点において想定される内容**

本研究で得られた試料・情報等を用いて、心筋梗塞患者に対する亜鉛補充療法に関する検討を予定しているが、その詳細についてはまだ決まっていない。新たに企画された研究を実施する前には必ず昭和大学横浜市北部病院の臨床試験審査委員会に研究実施申請を行ない、審査を受ける。本研究の同意において研究実施後の試料・情報の保管・使用に関する同意を取得した後においても、提供者本人からの同意撤回があれば直ちに本人の意向に沿って試験に伴う試料・情報を廃棄する。

**24モニタリング及び監査を実施（指針 第20の規定）する場合には、その実施体制及び**

**実施手順**

侵襲を伴う研究ではなく介入を行わないため該当せず。
